# Post-translational hydroxylation by 2OG/Fe(II)-dependent oxygenases as a novel regulatory mechanism in bacteria

**DOI:** 10.3389/fmicb.2014.00798

**Published:** 2015-01-15

**Authors:** Laura M. van Staalduinen, Zongchao Jia

**Affiliations:** Department of Biomedical and Molecular Sciences, Queen’s UniversityKingston, ON, Canada

**Keywords:** 2-oxoglutarate/Fe(II)-dependent oxygenase, post-translational hydroxylation, ribosomal oxygenase (ROX), prokaryote, YcfD

## Abstract

Protein hydroxylation has been well-studied in eukaryotic systems. The structural importance of hydroxylation of specific proline and lysine residues during collagen biosynthesis is well established. Recently, key roles for post-translational hydroxylation in signaling and degradation pathways have been discovered. The function of hydroxylation in signaling is highlighted by its role in the hypoxic response of eukaryotic cells, where oxygen dependent hydroxylation of the hypoxia inducible transcription factor both targets it for degradation and blocks its activation. In contrast, the role of protein hydroxylation has been largely understudied in prokaryotes. Recently, an evolutionarily conserved class of ribosomal oxygenases (ROX) that catalyze the hydroxylation of specific residues in the ribosome has been identified in bacteria. ROX activity has been linked to cell growth, and has been found to have a direct impact on bulk protein translation. This discovery of ribosomal protein hydroxylation in bacteria could lead to new therapeutic targets for regulating bacterial growth, as well as, shed light on new prokaryotic hydroxylation signaling pathways. In this review, recent structural and functional studies will be highlighted and discussed, underscoring the regulatory potential of post-translational hydroxylation in bacteria.

## INTRODUCTION

Of the commonly observed post-translational modifications, post-translational hydroxylation represents the smallest change. However, despite its diminutive nature, this modification may have significant effects on protein production and we are just beginning to discover its potential as a regulatory mechanism. Since the discovery of enzyme-catalyzed hydroxylation of prolyl residues during collagen biosynthesis, the importance of post-translational hydroxylation of proteins has been well established (Stetten, 1949; Hutton et al., 1966). More recently roles for protein hydroxylation in cell signaling and degradation pathways have been identified, expanding the significance of this post-translational modification. While important roles for protein hydroxylation have been observed, in comparison with other post-translational modifications such as phosphorylation, their full extent has yet to be determined (Loenarz and Schofield, 2011). Although not likely to be as widespread as others, new functions of protein hydroxylation are being discovered that indicate it may have a larger role than previously thought.

**FIGURE 1:**
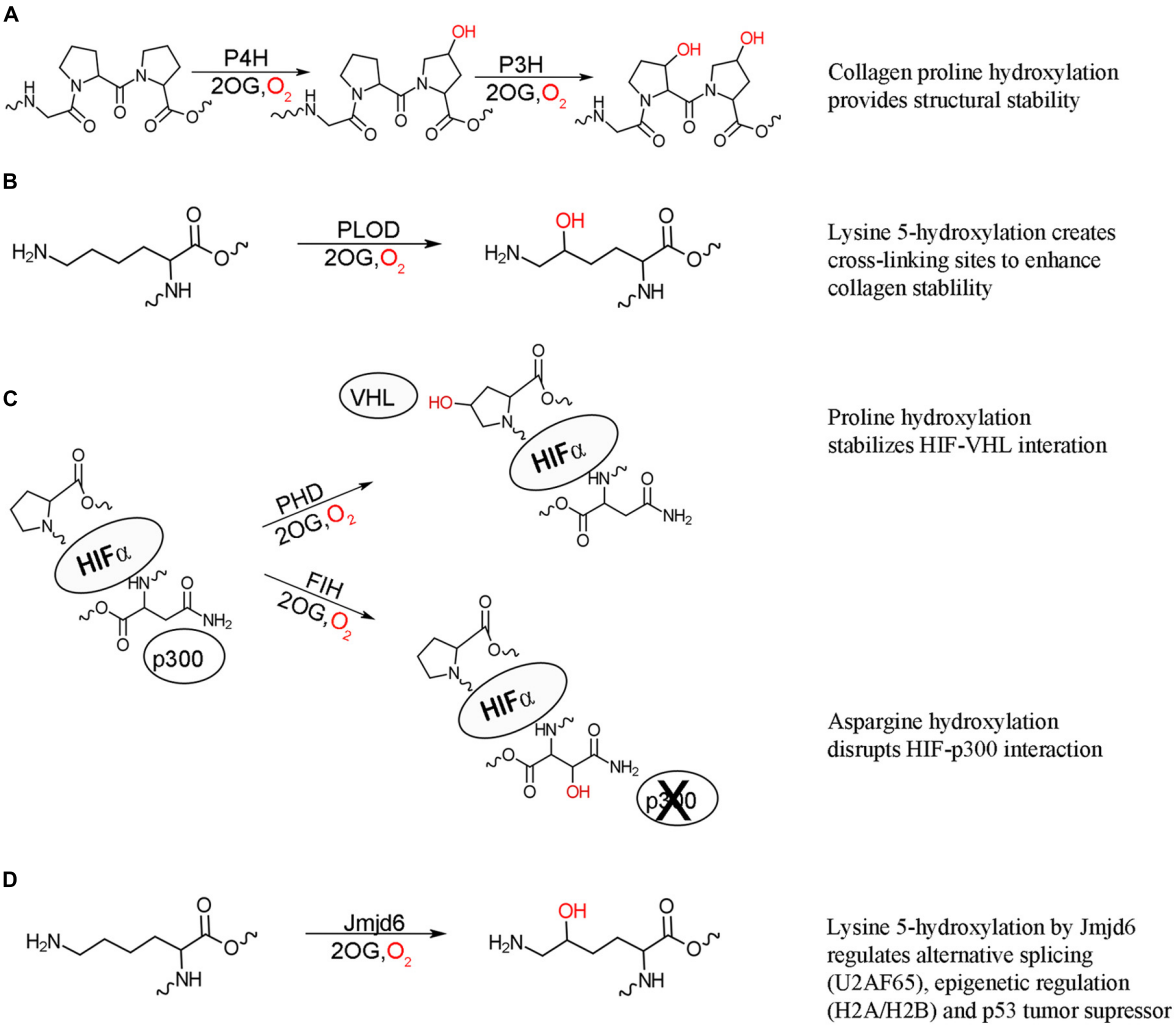
**2-oxoglutarate oxygenase catalyzed hydroxylation and their biochemical effects. (A)** Hydroxylation of proline residues of collagen by procollagen prolyl 4-hydroxylation (P4H) and prolyl 3-hydroxylase (P3H) increase the structural stability of collagen. **(B)** Hydroxylation of collagen lysine residues by procollagen lysine 5-hydroxylase (PLOD) provides further stability through creating cross-linking sites. **(C)** Hydroxylation of HIF proline residues by prolyl hydroxylase domain (PHD) enzymes stabilizes the interaction between HIF and von Hippel Lindau (VHL) protein targeting HIF for degradation, while asparaginyl hydroxylation of HIF by factor inhibiting HIF (FIH) acts to inhibit transcriptional activity by disrupting the interaction of HIF with p300. Both hydroxylation events result in suppression of the hypoxic response. **(D)** Lysyl hydroxylation of a variety of proteins by hydroxylase Jmjd6 has effects on alternative splicing (U2AF65), epigenetic regulation (histones H2A/H2B and H3/H4), and p53 tumor suppressor activity.

The post-translational hydroxylation of collagen, one of the most abundant structural proteins in animals, has been extensively studied. The discovery that the hydroxylation of prolyl and lysyl residues in collagen as a result of an oxygenase catalyzed modification provided the first evidence of the importance of enzyme catalyzed post-translational hydroxylation (Stetten, 1949; Hutton et al., 1966; Jenkins and Raines, 2002). Three different post-translational hydroxylations are present in collagen: 4*R*-hydroxy-*L*-proline, 3*S*-hydroxy-*L*-proline and 5*R*-hydroxy*-L-*lysine, with the 4*R*-hydroxy-*L*-proline being the most commonly observed (Myllyharju and Kivirikko, 2001). These hydroxylations are vital for the structure of collagen, which is comprised uniquely of three left-handed helices wound together around a central-axis to form a triple stranded right-handed tertiary structure, with multiple collagen molecules cross-linked to form connective tissues. The stability and strength of this structure is dependent upon a Gly-Xaa-Yaa repeating motif, where Xaa is typically *L-*proline and Yaa is typically 4*R*-hydroxy-*L*-proline (Engel and Bächinger, 2005). The hydroxylation of proline residues in collagen is catalyzed by procollagen prolyl 3- and 4-hydroxylases (P3H and P4H) to form the 3*S*-hydroxy-*L*-proline and 4*R*-hydroxy-*L*-proline, respectively (**Figure 1A**; Myllyharju, 2003; Vranka et al., 2004). The hydroxylated 4*R*-hydroxy-*L*-proline in the Yaa position is critical for stabilizing the structure, partly through essential hydrogen bonds and partly by the stereoelectric gauche effect (Prockop and Kivirikko, 1995; Myllyharju and Kivirikko, 2004). In contrast to the stabilizing effect of the 4*R*-hydroxy-*L*-proline, the rare 3*S*-hydroxy-*L*-proline has a less defined role, with initial evidence indicating a slight destabilizing effect (Jenkins et al., 2003). Evidence has now been found that this modification mediates inter-helical interactions and helps the assembly of the collagen triple helix (Weis et al., 2010). Mature collagen molecules are assembled by tissue specific cross-linking of domains flanking the triple stranded helical domain. This cross-linking is mediated by the presence of hydroxylysine, the formation of which is catalyzed by procollagen lysl 5-hydroxylase (PLOD; **Figure 1B**; Yamauchi and Sricholpech, 2012). Defects in all three types of collagen hydroxylation have been linked to number of diseases, highlighting the importance of this post-translational modification.

Post-translational hydroxylation is now known to be involved, not only in protein structural stability, but also in cellular signaling. The transcription factor hypoxia inducible factor (HIF) is critical for the initiation of the hypoxic response, which occurs when multicellular organisms are subjected to low oxygen levels (Palmer and Clegg, 2014). HIF is a constitutively expressed, heterodimeric protein, comprised of HIF1α and HIF1β subunits (Wang et al., 1995). The HIF1β subunit resides in the nucleus while, under hypoxic conditions, the HIF1α subunit is translocated into the nucleus and the active heterodimer recruits the transcriptional coactivator p300, initiating transcription of genes required for hypoxic response (reviewed in Hewitson and Schofield, 2004). Under normal oxygen levels, the HIF1α subunit is not detectable in the cell implicating oxygen regulated suppression of HIFα lifetime and activity in the cell (Huang et al., 1996). It is now known that post-translational hydroxylation occurs at three separate sites on HIF1α under normoxic conditions. The oxygen dependent post-translational hydroxylation of two critical proline residues by a family of three closely related prolyl hydroxylases (PHD1-3) results in the recruitment of HIF1α to the E3 ubiquitin ligase complex and subsequent degradation of HIF1α (**Figure 1C**; Ivan et al., 2001; Jaakkola et al., 2001; Masson et al., 2001; Yu et al., 2001). Another level of HIF inhibition under normal oxygen levels is the post-translational hydroxylation of an asparaginyl residue by the hydroxylase: factor inhibiting HIF (FIH). This hydroxylation blocks the interaction of HIF1α with p300, thus inhibiting transcriptional activity (**Figure 1C**; Lando et al., 2002). This hydroxylase-mediated control of the hypoxic response is the first comprehensively described instance of post-translational hydroxylation as a regulatory mechanism.

Following the discovery that HIF activity is regulated by hydroxylation, the possibility of similar systems being regulated by hydroxylation was investigated and revealed post-translational hydroxylation of lysine residues in the splicing factor U2 small nuclear ribonucleoprotein auxiliary factor 65-kDa subunit (U2AF65) catalyzed by the FIH related hydroxylase Jumonji domain 6 protein (Jmjd6; Webby et al., 2009). Jmjd6 was originally identified as a histone arginine demethylase (Chang et al., 2007); however, large scale analysis of Jmjd6 interacting proteins revealed that its predominate function is lysyl hydroxylation (**Figure 1D**; Webby et al., 2009). Hydroxylation of U2AF65 was found to change alternative RNA splicing for some genes indicating a role in the regulation of gene splicing, with knockdown of Jmjd6 resulting in similar splicing patterns to those observed under hypoxic conditions (Webby et al., 2009). Jmjd6 is now known to regulate alternative gene splicing through interaction with a number splicing factors, as well as the pre-RNA itself, though the exact conditions and physiological role for hydroxylation have yet to be identified (Heim et al., 2014). Jmjd6 was subsequently found to also hydroxylate lysine residues on the histones H2A/H2B and H3/H4 (Unoki et al., 2013). The hydroxylated lysines were found to inhibit both *N-*acetylation and *N-*methylation of histone peptides *in vitro*. Conversely, both *N-*acetylation and *N-*methylation of lysine residues blocked Jmjd6 catalyzed hydroxylation. Combined these results suggest a role of post-translational histone hydroxylation in the epigenetic regulation of gene expression and chromosomal rearrangement. Most recently, Jmjd6 was found to catalyze the lysyl hydroxylation of the tumor suppressor p53, decreasing p53 activity, and promoting colon carcinogenesis (Wang et al., 2014). Jmjd6 hydroxylation of p53 reveals a connection between oxygen levels, cell cycle control, and apoptosis.

## PROTEIN HYDROXYLASES

Post-translational hydroxylation involves the oxidative conversion of a C–H bond to a C–OH group on an amino acid side chain. Protein hydroxylases, the enzymes responsible for catalyzing this conversion, are found to belong to the 2-oxoglutarate (2OG)/Fe^2+^-dependent oxygenase (2OG oxygenase) superfamily of proteins (Loenarz and Schofield, 2011). The majority of these enzymes use a Fe^2+^ cofactor and 2OG and dioxygen as co-substrates. 2OG oxygenases are widely distributed evolutionarily conserved enzymes involved in many biologically important processes such as DNA repair, protein modification, lipid metabolism, and secondary metabolite production in plants and microbes. The enzymes catalyze a diverse array of oxidative reactions, including desaturation, ring formation or expansion, epimerization, and carbon–carbon bond cleavage. However, the most common reaction they catalyze is hydroxylation (Hausinger, 2004). 2OG oxygenases are known to catalyze the hydroxylation of a variety of substrates ranging from small molecules to macromolecular molecules, including both proteins and DNA.

**FIGURE 2:**
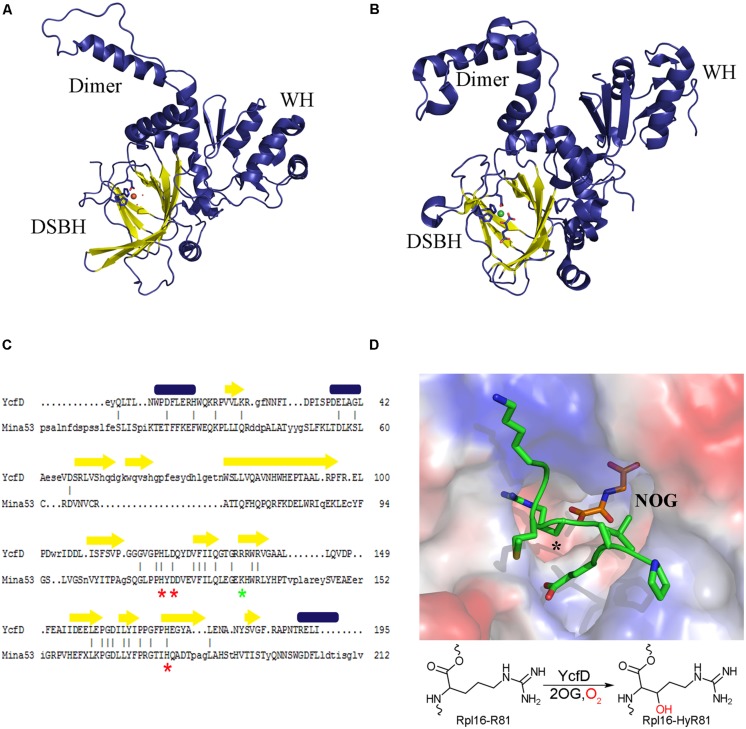
**Structure conservation of ROX enzymes.** A structural comparison of **(A)** the bacterial ROX YcfD (PDB ID 4NUB) to **(B)** the eukaryotic ROX Mina53 (PDB ID 4BU2). The structures align with an overall RMSD of 2.6 Å. The characteristic DSBH is shown in yellow, and the dimerization domain (dimer) and the winged-helix domain (WH) are labeled. **(C)** Structure based sequence alignment of the DSBH from YcfD and Mina53. Conserved metal binding residues are marked with a red asterisk and the 2OG binding residues are marked with a green asterisk. Alignment was done using Dalilite (Holm and Park, 2000). **(D)** Binding of YcfD to Rpl16 peptide. The surface of the Rhodothermus marinus YcfD (PDB ID 4CUG) active site is shown with an Rpl16 peptide (green) and the 2OG analog N-oxalylglycine bound (NOG; orange). The site of hydroxylation is marked with an asterisk (*). Below, a schematic hydroxylation of Rpl16-R81 by YcfD is shown.

Proteins belonging to the 2OG oxygenase family are identified by a conserved HXD/EX_n_H sequence motif, where the histidine and aspartate/glutamate residues are involved in coordinating the metal cofactor (Clifton et al., 2006). The three-dimensional structures of many 2OG oxygenases, including a number of post-translational hydroxylases have been determined. All 2OG oxygenases are found to have a conserved core structure of eight β-strands, which form two anti-parallel β-sheets that come together in a right-handed double-stranded β-helix (DSBH) or jelly roll (**Figure 2**; Roach et al., 1995; Clifton et al., 2006). The conserved sequence motif is found within the DSBH, and marks the location of the enzyme active site where Fe^2+^ is brought together with the substrates. The DSBH forms a very robust active site allowing for the accommodation of the different substrates of the 2OG oxygenases, and for the catalysis of complex oxidative reactions. Structural data, combined with kinetic and spectroscopic analysis suggest that the 2OG oxygenases share a common enzymatic mechanism wherein the Fe^2+^-bound enzyme interacts with 2OG, triggering reaction with dioxygen, which leads to the formation of a ferryl intermediate that acts as a reactive oxidizing species formed upon oxidative decarboxylation of 2OG (Holme, 1975; Chowdhury et al., 2014). The ferryl intermediate is then poised to react with the substrate. This is where the mechanisms diverge depending on the type of reaction being catalyzed, accounting for the breadth, and versatility of this class of enzyme.

## RIBOSOMAL OXYGENASES

With the discovery of the importance of hydroxylation of HIF and splicing relating proteins by 2OG oxygenases in eukaryotes, the question of whether oxygenase catalyzed post-translational hydroxylation has a role in prokaryotic cells was raised. The *Escherichia coli* gene of unknown function, *ycfD,* was identified as a potential 2OG oxygenase, which was confirmed by the observation of YcfD bound to the 2OG as a co-substrate (van Staalduinen et al., 2014) and catalyzed 2OG turnover in the absence of substrate (Ge et al., 2012). A peptide screen combined with co-immunoprecipitation analyses revealed that YcfD hydroxylated the β carbon of arginine 81 of ribosomal protein L-16 (Rpl16; Ge et al., 2012). Interaction with Rpl16 was independently confirmed and shown to be highly specific by glutathione *S*-transferase pull-down experiments (van Staalduinen et al., 2014). Consistent with the close link between translation and growth, alteration of YcfD expression has been shown to have dramatic effects on cell growth. Comparison of wild-type cell growth to that of a strain lacking the *ycfD* gene (Δ*ycfD*) showed that, under normal conditions, there was no difference between the two cell lines. However, under nutrient-limiting conditions, the growth of Δ*ycfD* cells was significantly reduced, which correlated with a reduction in bulk protein translation by three- and fourfold (Ge et al., 2012). Overexpression of YcfD was also shown to significantly inhibit *E. coli* colony formation under standard growth conditions, indicating a clear role for YcfD in *E. coli* cell growth regulation (van Staalduinen et al., 2014). Two human homologs to YcfD, Mina53 and NO66, were also identified to hydroxylate ribosomal proteins, and have similar effects on cell proliferation (Tsuneoka et al., 2002; Teye et al., 2004; Zhang et al., 2005; Suzuki et al., 2007; Ge et al., 2012). Together, Ycfd, Mina53, and NO66 are the founding members of a novel class of evolutionarily conserved ribosomal oxygenases (ROXs).

Structural studies of YcfD and other ROX enzymes showed that they are comprised of three domains: an N-terminal DSBH, followed by a dimerization domain and a C-terminal winged-helix domain (WH; **Figures 2A,B**; Chowdhury et al., 2014; van Staalduinen et al., 2014). The N-terminal DSBH displays the characteristic topology of a stereotypical 2OG oxygenase. Despite overall low sequence homology (15% homology to Mina53 and NO66) YcfD is structurally very similar to the eukaryotic ROX enzymes (YcfD-Mina53 RMSD 2.6 Å). The DSBH is particularly well conserved with residues involved in metal and 2OG binding conserved (**Figure 2C**), while the dimerization and WHs show much lower conservation. The ROX active site is found in a pocket within the DSBH, and the substrate of YcfD, Rpl16 was shown to dock to the active site in a complementary manner (van Staalduinen et al., 2014). Co-crystallization of peptide substrates with the ROX enzymes provides more detailed insight into the interaction (Chowdhury et al., 2014). There are very minor changes observed when the Rpl16 peptide is bound by YcfD; the overall structure remains largely unchanged with only a few residues in the active site shifting to accommodate the substrate. The arginine side chain to be hydroxylated sits deep in the active site with the β-carbon aligned with 2OG, in an ideal geometry for hydroxylation (**Figure 2D**). The surface of the area surrounding the active site of the YcfD is intimately involved with binding of the substrate with a number of clefts to allow docking of side chains to the surface of the enzyme. The dimerization domain is comprised of three α-helices which form intimate contacts with the dimerization domain of another molecule and have been shown to be important for catalytic activity (Chowdhury et al., 2014). The C-terminal WH distinguishes the ROX proteins from other 2OG oxygenases. Typically, WHs mediate protein–protein or protein–nucleic acid interactions (Teichmann et al., 2012); in this case, it is unlikely that the ROX proteins bind nucleic acids directly due to the overall negative charge of this domain (Chowdhury et al., 2014). Instead, it is likely that this essential domain plays a role in substrate binding, either binding substrate directly or interacting with another part of the ribosomal complex.

## REGULATORY POTENTIAL OF ROX IN PROKARYOTES

The substrate of YcfD, Rpl16, is an essential late-assembly component of the 50S ribosomal subunit and is responsible for the architectural organization of the aminoacyl-tRNA binding site (Nierhaus, 1991). A loss of Rpl16 has been associated with defects in stages of both ribosomal assembly and function, including maturation of the 50S subunit (Jomaa et al., 2014), binding of the 30S subunit (Kazemie, 1975), association with aminoacyl-tRNA (Kazemie, 1976), peptidyl-tRNA hydrolysis activity (Tate et al., 1983), peptidyl transferase activity (Moore et al., 1975; Hampl et al., 1981), as well as antibiotic interactions (Nierhaus and Nierhaus, 1973; de Bethune and Nierhaus, 1978; Teraoka and Nierhaus, 1978). The structure of Rpl16 has been determined by NMR (PDB ID: 1WKI), revealing that the hydroxylation site is on an extended, flexible loop that becomes locked upon binding to the 23S rRNA, as observed in crystal structures of the bacterial ribosome (Harms et al., 2001; Nishimura et al., 2004; Dunkle et al., 2010). The site of YcfD hydroxylation, R81, is inserted between two 23S rRNA helices in the intact ribosome, indicating a role for the hydroxyl group in stabilizing the architecture of the aminoacyl-tRNA binding site through hydrogen bonding and, ultimately, in the spatial optimization of the Rpl16-rRNA complex. There is evidence that YcfD binds very specifically to Rpl16, and is capable of pulling down Rpl16 in the absence of other ribosomal proteins. In addition, Rpl16 plays a role as a late ribosomal assembly protein, which indicates a potential function for YcfD in sequestering Rpl16 prior to its addition to the maturing ribosome, thus ensuring proper assembly of the ribosome. The overall importance of Rpl16 in the competency of the bacterial ribosome, combined with the fact that it is the target of a number of antibiotics, indicate that hydroxylation of Rpl16 by YcfD may play a role in the regulation of protein translation and, consequently, in bacterial cell growth.

2-oxoglutarate oxygenases have been found to provide a link between metabolism and transcriptional regulation via evidence that oxygenases involved in transcriptional regulation are inhibited by increased amounts of tricarboxylic acid cycle intermediates or 2-hydroxyglutarate in tumor cells (Ge et al., 2012; Mullen and DeBerardinis, 2012). A similar relationship between metabolism and translation through regulation of ROX activity may exist and investigation into the effects of metabolic molecules on ROX activity could lead to an understanding of this relationship. This connection between metabolism and translational regulation seems very intuitive, particularly for bacteria, as cell growth would need to decrease in response to limited nutrition and, conversely, under nutrient rich conditions the cells do not need to limit their growth. The activity of the ROX enzymes, like that of other 2OG oxygenases, was also found to be limited under hypoxic conditions (Ge et al., 2012). This loss of YcfD activity under anaerobic conditions suggests a regulatory role for the hydroxylase under hypoxic stress, resulting in reduced translation and subsequent loss of cell growth. As the connection between ROX enzyme activity and cell growth is better understood, there is opportunity for the development of new antibiotics which target YcfD, the YcfD-Rpl16 complex, or hydroxylated Rpl16.

## CONCLUDING REMARKS

Post-translational hydroxylation, though well characterized in eukaryotes, remains understudied in prokaryotes. The discovery that YcfD is a bacterial ROX, responsible for the hydroxylation of an essential component of the bacterial ribosome, highlights the potential for post-translational hydroxylation as an important bacterial regulatory mechanism. The sensitivity of hydroxylases to alterations in metabolism and hypoxic conditions makes them ideal candidates for regulating bacterial cell response to changes in the environment. Investigation of other putative 2OG oxygenase could elucidate novel post-translational hydroxylation regulatory pathways in prokaryotes, as well as uncover novel therapeutic targets.

## Conflict of Interest Statement

The authors declare that the research was conducted in the absence of any commercial or financial relationships that could be construed as a potential conflict of interest.
